# Red Blood Cell Distribution Width (RDW) as Predictor of the Clinical Course and Mortality in Patients With Acute Respiratory Distress Syndrome (ARDS): A Retrospective Study

**DOI:** 10.1002/hsr2.71392

**Published:** 2025-10-28

**Authors:** Anna Kirsch, Felix Niebhagen, Sandra Waske, Fabian Schröer, Victoria Buenger, Oliver Hunsicker, Steffen Weber‐Carstens, Jan Adriaan Graw, Mario Menk

**Affiliations:** ^1^ Department of Anesthesiology and Critical Care Medicine University Hospital Carl Gustav Carus and Carl Gustav Carus Faculty of Medicine, TU Dresden Dresden Germany; ^2^ Department of Anesthesiology and Critical Care Medicine University Hospital Berlin, Charité – Universitätsmedizin Berlin Berlin Germany; ^3^ Department of Anesthesiology University Hospital Erlangen, Universität of Erlangen Ulm Germany

**Keywords:** acute respiratory distress syndrome, intensive care unit, mortality, prognosis, prognostic marker, red cell distribution width

## Abstract

**Background and Aims:**

Evidence on the prognostic value of red blood cell distribution width (RDW) in critically ill patients with acute respiratory distress syndrome (ARDS) is limited. This study evaluated RDW as a predictor of mortality in patients with ARDS.

**Methods:**

A retrospective study conducted at an ARDS referral center included 1037 adult ICU patients (2007–2019). RDW values at ICU admission and during ICU stay were evaluated for their ability to predict mortality using statistical analyses, including logistic regression, Cox regression, and receiver operating curve analysis, and further specified according to Youden's method.

**Results:**

In total, 1037 ICU patients with ARDS were included in the analysis. Non‐survivors had significantly higher RDW on ICU admission than survivors (survivors' median RDW 15.4% [14.2; 17.0%] vs. non‐survivors' median RDW 16.55% [15.2; 18.0%]; *p* < 0.0001). A cut‐off RDW ≥ 16.4% at ICU admission effectively stratified risk and was associated with higher SAPS II and APACHE II scores, prolonged mechanical ventilation, longer ICU stay, and higher ICU mortality. Patients with RDW ≥ 16.4% had almost double the risk of ICU death compared to those with lower values (hazard ratio: 1.96; 95% CI: 1.61–2.40; *p* < 0.001). Median survival was significantly lower in patients with RDW ≥ 16.4% (27 vs. 72 days; log rank, *p* < 0.0001). RDW values corresponded to ARDS severity and reflected worse clinical outcomes.

**Conclusion:**

RDW at ICU admission predicts mortality and clinical course in ARDS patients. RDW may serve as a reliable marker of ARDS severity and mortality risk in the ICU.

**Abbreviations**
APACHE: acute physiology, age, chronic health evaluation
ARDS: acute respiratory distress syndrome
AUC: area under the curve
CI: confidence interval
COPD: chronic obstructive pulmonal disease
ECMO: extracorporeal membrane oxygenation
FiO_2_
: the inspiratory fraction of oxygen
HR: hazard ratio
ICU: intensive care unit
PaO_2_
: arterial pressure of oxygen
PEEP: positive end‐expiratory pressure
Pmax: maximal pressure
RDW: red blood cell distribution width
ROC: receiver operating curve
SAPS: simplified acute physiology score
SOFA: sepsis‐related organ failure
TISS 28: Therapeutic Intervention Scoring System

## 1 Introduction

Acute respiratory distress syndrome (ARDS) is a severe, life‐threatening condition characterized by the rapid onset of respiratory failure due to diffuse alveolar damage and increased pulmonary vascular permeability. It is defined by the Berlin criteria, which include an acute onset within 1 week of a known clinical insult; bilateral opacities on chest imaging that cannot be fully explained by effusions or lung collapse; and hypoxemia with a PaO_2_/FiO_2_ ratio ≤ 300 mmHg under positive end‐expiratory pressure (PEEP). ARDS is classified as mild, moderate, or severe based on the degree of hypoxemia. Diagnosis is clinical and radiological, with cardiac failure or fluid overload being excluded as the primary cause. Treatment is primarily supportive, involving lung‐protective mechanical ventilation. Adjunctive therapies include prone positioning, neuromuscular blockade, and, in select cases, extracorporeal membrane oxygenation (ECMO) [1, 2]. Despite recent advances in supportive care and mechanical ventilation, mortality rates in patients with ARDS remain high, ranging from 30% up to 50% [1, 3]. Due to the complexity and expense of supportive therapies, such as veno‐venous ECMO, there is a certain need for improved prognostic markers to identify patients at risk of severe organ failure and mortality. These markers could support clinical decision‐making and facilitate the early referral of patients to specialized ARDS/ECMO centers.

Several prognostic markers have been proposed for ARDS, including clinical variables such as age, comorbidities, severity of illness scores, and biomarkers of inflammation such as interleukin‐6, C‐reactive protein, and procalcitonin [4, 5, 6, 7, 8]. While these markers have shown some promise in predicting outcomes in ARDS, they have limitations in terms of their specificity and sensitivity [8].

Recent studies have suggested that the red blood cell distribution width (RDW), a measure of the variability in the size of red blood cells, may be a useful prognostic marker in critically ill patients, including those with ARDS [9, 10, 11, 12, 13]. In healthy adults, RDW ranges from 11.5% to 14.5% and is usually included in the routine complete blood count measurements [14]. RDW has traditionally been used as an indicator of anemia, but recent evidence suggests that it may also be associated with inflammation, oxidative stress, and cardiovascular disease [9, 10, 11, 12]. However, some studies have linked high RDW values with increased mortality rates in critically ill patients [15, 16]. The underlying mechanisms for the association between RDW, severity of illness, and mortality are not well understood, but may involve inflammation and impaired oxygen delivery [5, 17, 18]. Therefore, RDW has received some attention as a possible new, inexpensive, and rather simple diagnostic and prognostic marker for patients with acute lung failure, but a clear cut‐off value for patients with ARDS of all types remains elusive [19].

Given its routine availability and low cost as part of standard blood tests, RDW represents a readily accessible marker that could enhance early risk stratification in ARDS patients. Its potential association with key pathological processes such as inflammation and impaired oxygenation highlights its clinical relevance in monitoring disease progression and guiding therapeutic decisions. Therefore, the main objective of this study was to investigate the prognostic value of RDW regarding the clinical course and mortality in critically ill patients with ARDS.

## 2 Materials and Methods

### 2.1 Setting and Patients

This retrospective study was conducted at a single ARDS referral center of the Department of Anesthesiology and Intensive Care Medicine, Charité – Universitätsmedizin Berlin. The study was approved by the local ethics committee of the Charité – University Medicine Berlin (EA2/172/17). All adult patients admitted with ARDS according to the Berlin definition between January 2007 and December 2019 were included in the study. Patients under 18 years of age, patients who were readmitted after discharge, and patients who died within the first 6 h after intensive care unit (ICU) admission were excluded from the analyses. Patients for whom RDW values were not available were also excluded. Treatment of ARDS followed local standard operating procedures, including lung protective mechanical ventilation and rescue therapies such as prone positioning, treatment with inhaled nitric oxide, or therapy with ECMO.

### 2.2 Data Collection

Data were extracted from the electronic patient data management systems (COPRA, Sasbachswalden, Germany; SAP, Walldorf, Germany) as previously described [20]. In addition to basic demographic data, ICU admission scores (Simplified Acute Physiology Score [SAPS II], Sepsis‐related Organ Failure [SOFA], Acute Physiology and Chronic Health Evaluation [APACHEII], and Therapeutic Intervention Scoring System [TISS‐28]) were assessed. The severity of ARDS was classified according to the Berlin definition [1]. Etiologies of ARDS, such as pneumonia, sepsis of extrapulmonary origin, trauma, aspiration, and others, were determined. Parameters of pulmonary gas exchange and mechanical ventilation, such as peak inspiratory pressure, mean airway pressure, PEEP, the inspiratory fraction of oxygen (FiO_2_), arterial partial pressure of oxygen (PaO_2_) and pulmonary compliance were recorded. Other variables collected were: use, settings, and duration of ECMO, the duration of mechanical ventilation, ICU length of stay, and all‐cause ICU mortality.

### 2.3 Laboratory Tests

RDW was routinely measured in patients' red blood cell counts during their ICU stay and was calculated as the standard deviation of the red blood cells divided by the mean corpuscular volume. RDW is a measure of the variability of red blood cell size and is expressed as a percentage [12]. In instances where multiple RDW measurements were recorded on a daily basis, the maximum value was utilized for the analysis, as this was deemed to be the most reliable indicator of disease severity. RDW values at ICU admission for each patient were used for receiver operating curves (ROC), linear regression, Cox regression, and survival analysis. The RDW was determined by the local laboratory using a Sysmex XN‐1000 analyzer. An RDW range between 11.5% and 14.5% was considered normal for grouping patients.

### 2.4 Endpoints

The primary endpoint was all‐cause mortality in the ICU after admission. Secondary endpoints included RDW change over time, ICU length of stay, gas exchange and acid–base status variables, and “failure‐free days” – composites such as ECMO‐free, ventilator‐free, sedation‐free, organ dysfunction‐free, renal replacement therapy (RRT)‐free, and vasopressor‐free days. “Failure‐free days” composites were defined and analyzed as previously described [20].

### 2.5 Statistical Analysis

Discrete variables are presented as absolute numbers, counts, or percentages as indicated. Continuous variables are reported as medians with 25; 75 percentiles. For patient demographic characteristics, statistical differences between groups were assessed using Fisher's exact test for categorical variables or the Mann–Whitney *U*‐test for continuous variables, as appropriate. Predictive validity for mortality was assessed using ROC and corresponding results for the area under the curve (AUC). In addition, a cut‐off value for mortality based on RDW was determined using Youden's method (representing the highest sum of sensitivity and specificity) as described previously [21]. This cutoff value was used to assign patients to one of two groups (i.e., above or below the calculated cutoff value) for subsequent predictive analyses.

For multivariable analysis, we performed logistic regression using GraphPad Prism 10. Variables included in the multivariable model were selected based on their statistical significance in univariate analysis (*p* < 0.05) and clinical relevance. A backward elimination procedure was applied, in which variables were removed sequentially based on their *p*‐values. As Prism 10 does not support model selection based on information criteria such as AIC or BIC, the selection process was guided by significance levels and clinical judgment. Model fit and assumptions were assessed accordingly. Proportional hazards assumptions and collinearity diagnostics were automatically assessed by GraphPad Prism 10 during the regression analyses. No violations or significant collinearity were detected. Kaplan–Meier estimates were used to visualize differences in survival between patients using the calculated cutoff value. Differences in survival between patients below or above the calculated cutoff value were tested using the log‐rank test. Multivariate Cox regression with stepwise backward selection was used to test for factors influencing survival, including variables that showed a statistically significant effect in the univariate analyses. Statistical analyses were performed using IBM SPSS Statistics, Version 24 (SPSS, Chicago, IL, USA) and R Project for Statistical Computing, Version 3.4.0 (2017‐04‐21), Copyright 2017, The R Foundation for Statistical Computing, and GraphPad PRISM version 10 (San Diego, CA, USA). A two‐tailed *p*‐value < 0.05 was considered statistically significant. All tests should be considered as exploratory analysis, so no adjustments were made for multiple testing.

## 3 Results

### 3.1 Patient Characteristics

In total, 1044 critically ill patients with ARDS were admitted between January 2007 and December 2019, of whom 1037 had complete RDW data, fulfilled entry criteria, and were included in the analysis. The majority of patients (94.3%) had elevated RDW values above the local laboratory reference value of 14.5%. Acute clinical risk scores, such as APACHE II and SAPS II, were high in ARDS patients on admission to the ICU, indicating severe critical illness. According to the Berlin definition, Most patients had severe ARDS (*n* = 848; 81.8%). The main cause for ARDS in this study was pneumonia (*n* = 669; 64.5%), followed by sepsis of extrapulmonary origin (*n* = 204; 19.7%). Overall, ICU mortality was 39.4%. Characteristics of the study population are shown in Table 1.

**Table 1:** Patient characteristics.

	All patients	RDW ≥ 16.4%	RDW < 16.4%	*p* value
*n*	1037	444 (42.8%)	593 (57.2%)	
Basic characteristics				
Age (years)	53 (40, 64)	53 (42, 65)	52 (39, 63)	0.04
Male sex (*n*)	677 (65.3%)	270 (60.8%)	407 (68.6%)	0.01
Body mass index (kg/m^2^)	26.23 (23, 31.22)	26.12 (22.86, 31.25)	26.3 (23.03, 31.22)	n.s.^a^
Pre‐existing conditions				
Diabetes mellitus Type 1 and 2 (*n*)	221 (21.3%)	102 (23.0%)	119 (20.1%)	n.s.^b^
Liver disease (*n*)	147 (14.2%)	85 (19.2%)	62 (10.5%)	< 0.0001
Malignancy (*n*)	234 (22.6%)	128 (28.8%)	106 (17.9%)	< 0.0001
Chronic kidney disease (*n*)	115 (11.1%)	64 (14.4%)	51 (8.6%)	0.0037
Heart failure (*n*)	66 (6.4%)	37 (8.3%)	29 (4.9%)	0.028
COPD (*n*)	175 (16.9%)	74 (16.7%)	101 (17.0%)	n.s.^b^
Severity of illness scores at ICU admission				
SAPS II	55 (39, 68)	58 (43, 73)	52 (38, 65)	< 0.0001
APACHE II	26 (19, 33)	28 (21, 35)	24 (18, 32)	< 0.0001
SOFA II	11 (9, 14)	12 (9, 15)	11 (8, 13)	< 0.0001
Etiology of ARDS				
Pneumonia (*n*)	669 (64.4%)	297 (66.7%)	372 (63.0%)	n.s.^b^
Sepsis (*n*)	204 (19.7%)	117 (26.5%)	87 (14.60%)	< 0.0001
Trauma (*n*)	51 (4.9%)	13 (2.9%)	38 (6.4%)	0.013
Others (*n*)	113 (11%)	38 (8.5%)	76 (12.7%)	< 0.05
Severity of ARDS according to the “Berlin definition of ARDS”				
Mild (*n*)	36 (3.5%)	13 (2.9%)	23 (3.9%)	n.s.^b^
Moderate (*n*)	153 (14.7%)	52 (11.7%)	101 (17%)	0.02
Severe (*n*)	848 (81.8%)	379 (85.4%)	469 (79.1%)	0.011
Pulmonary gas exchange and mechanical ventilation (average of the first 6 h after ICU admission)				
Pmax (cm H_2_O)	34 (30, 38)	35 (30, 39)	33 (30, 37)	0.002
PEEP (cm H_2_O)	16 (14, 19)	16 (14, 19)	16 (13, 19)	n.s.^a^
pH	7.32 (7.25, 7.39)	7.31 (7.23, 7.39)	7.32 (7.26, 7.39)	n.s.^a^
P/F ratio (Horowitz‐index)	144 (102, 213)	136 (97, 204)	150 (107, 217)	0.006
FiO_2_%	82 (70, 94)	84 (72, 95)	81 (67, 93)	0.009
PaO_2_ (mmHg)	112 (85, 152)	108 (83, 146)	116 (85, 158)	n.s.^a^
Pulmonary compliance (mL/cm H_2_O)	31 (21, 44)	27 (18, 38)	35 (25, 48)	< 0.0001
Mechanical ventilation (h)	387 (192.5, 691.5)	336.5 (124.8, 636)	407 (221, 716)	0.003
Extracorporeal lung support (ECLS)				
ECMO (*n*)	479 (46.2%)	221 (49.8%)	258 (43.5%)	n.s.^b^
Length of ECLS (h)	71 (0, 329.5)	93 (0, 335.5)	25 (0, 307)	0.047
Dialysis				
Dialysis (*n*)	596 (57.4%)	290 (65.3%)	306 (51.61%)	< 0.0001
Duration of dialysis during ICU stay (h)	60 (0, 230.5%)	80.5 (0, 248.3)	37 (0, 215)	0.001
ICU length of stay (days)	18 (9, 31)	15.5 (6.75, 30)	19 (10, 33)	0.003
ICU mortality	410 (39.5%)	239 (54.07%)	170 (28.57%)	< 0.0001

*Note:* Patients were grouped by RDW value at ICU admission as indicated. Data are expressed as medians (25%–75% quartiles) or frequencies (%), as indicated.

Abbreviations: APACHE, acute physiology and chronic health evaluation; ARDS, acute respiratory distress syndrome; COPD, chronic obstructive pulmonal disease; ECMO, extracorporeal membrane oxygenation; FiO_2_, fraction of inspired oxygen; ICU, intensive care unit; n.s., not significant; PaO_2_, arterial pressure of oxygen; PEEP, positive end expiratory pressure; Pmax, maximal pressure; SAPS, simplified acute physiology score; SOFA, sepsis‐related organ failure assessment.

^a^ Mann–Whitney *U*‐test.

^b^ Fisher's exact test.

### 3.2 RDW at ICU Admission, Severity of ARDS, and Mortality

**Figure 1:**
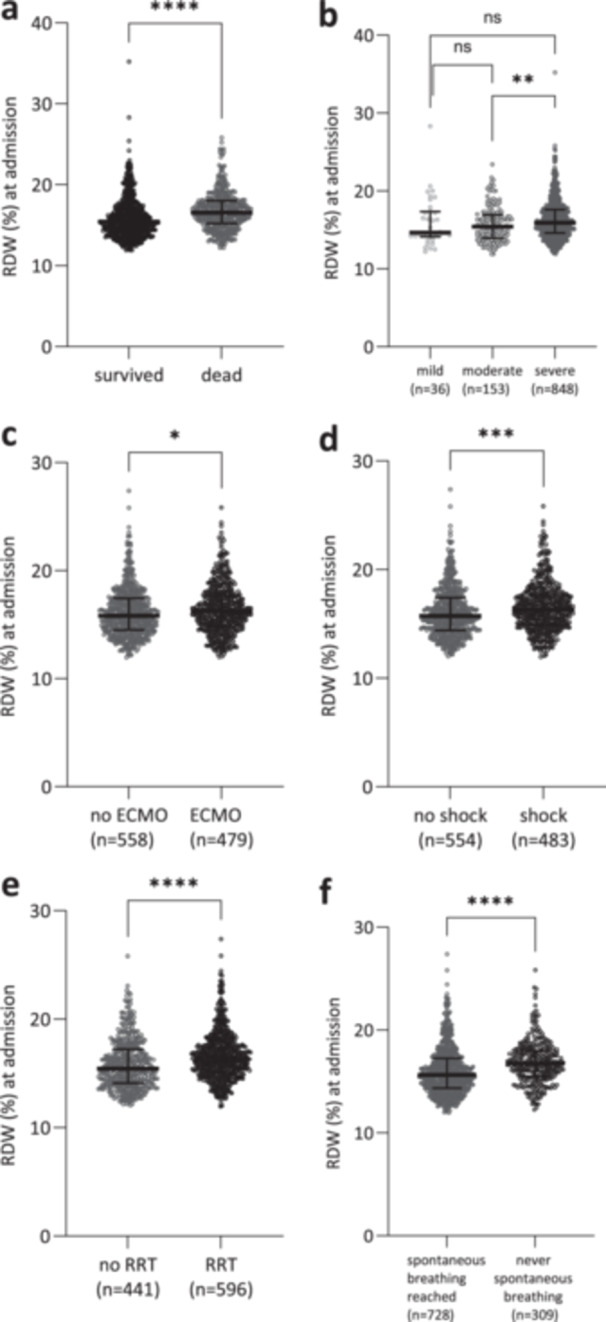
Maximum value of red cell distribution width (RDW) in critically ill patients with acute respiratory distress syndrome (ARDS) at ICU admission grouped by (a) survivors and non‐survivors; (b) severity of lung failure according to the Berlin definition of ARDS (mild, moderate, severe); (c) implementation of extracorporeal membrane oxygenation (ECMO) and no ECMO; (d) septic shock and no septic shock; (e) Implementation of renal replacement therapy (RRT) or no RRT; (f) reaching spontaneous breathing or not reaching spontaneous breathing. N.s. as indicated, Mann–Whitney test, *p < 0.05; ***p < 0.001; ****p < 0.0001; ns, not significant.

RDW values at ICU admission were found to be significantly associated with mortality in critically ill patients with ARDS. Overall, non‐survivors had significantly higher RDW values than survivors (survivors median RDW 15.4% [14.2; 17.0%] vs. non‐survivors median RDW 16.55% [15.2; 18.0%]; *p* < 0.0001; Figure 1a). The severity of lung failure corresponded with RDW values on admission to the ICU. When classified according to the Berlin definition of ARDS, patients with moderate and severe ARDS had significantly higher RDW values than patients with mild ARDS (mild ARDS median RDW 14.6% [14.1; 17.3%] vs. moderate ARDS median RDW 15.7% [13.9; 16.9%], ns.), (mild ARDS median RDW 14.6% [14.1; 17.3%] vs. severe ARDS median RDW 16.3% [14.6; 17.6%], *p* < 0.01), (moderate ARDS median RDW 15.7% [13.9; 16.9%], vs. severe ARDS median RDW 16.3% [14.6; 17.6%], *p* < 0.01; Figure 1b). In accordance, patients in need of ECMO had significantly higher RDW values at ICU admission (no ECMO median RDW 15.8% [11.9; 17.5%], vs. ECMO median RDW 16.2% [14.9; 25.9%], *p* < 0.05; Figure 1c). Significantly higher RDW values were also present in patients with shock, the need of RRT and in patients that never reached spontaneous breathing in the later course of ARDS (no shock median RDW 15.7% [14.4; 17.4%], vs. shock median RDW 16.3% [14.9; 17.6%], *p* < 0.01; Figure 1d; no RRT median RDW 15.4% [14.1; 17.2%], vs. RRT median RDW 16.3% [15; 17.7%], *p* < 0.01; Figure 1e; spontaneous breathing median RDW 15.6% [14.4; 17.2%], never spontaneous breathing median RDW 16.8% [15.5; 18%], *p* < 0.01; Figure 1f).

**Figure 2:**
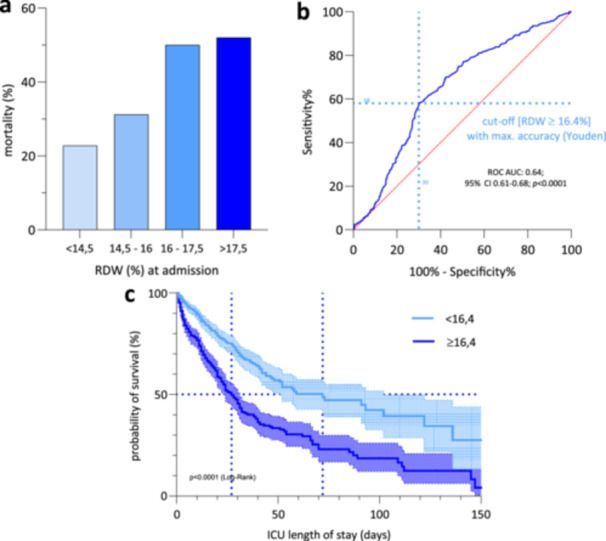
(a) ICU mortality rates in different RDW groups. ICU mortality of patients with ARDS in relation to the maximum RDW value at admission, *n* = 1037. (b) Cut‐off value by RDW value at admission. Receiver operator curve (ROC) for the determination of predictive validity of RDW measurements. The highest sum of sensitivity and specificity is used for calculating a cut‐off value. ROC AUC: 0.64; 95% CI: 0.61–0.68; *p* < 0.0001. (c) Survival grouped by RDW cut‐off value ≥ 16.4%. Probability of survival depicted as a Kaplan–Meier curve of patients with ARDS grouped by RDW cut‐off value of ≥ 16.4% from ROC analysis. Log‐rank test, *p* < 0.0001, *n* = 1037, median survival < 16.4%: 72 days, ≥ 16.4% 27 days.

Higher values of RDW at ICU admission were associated with higher mortality rates. Mortality rate was 22.8% in patients with normal RDW values at ICU admission, but increased to 52% when RDW values of more than 17.5% were present. (Figure 2a). According to Youden's method, a cut‐off for prediction of mortality based on RDW at ICU admission of 16.4% was found to stratify risk best. This cut‐off effectively divided the study population into two groups with the most significant difference in ICU mortality (ROC AUC: 0.64; 95% confidence interval [CI]: 0.61–0.68; *p* < 0.0001) (Figure 2b). ARDS patients below this RDW threshold level had a significant survival advantage with a median survival of 72 days compared to 27 days for the high RDW group (*n* = 1037, median survival < 16.4%: 72 days, ≥ 16.4% 27 days, log‐rank test *p* < 0.0001; Figure 2c). These patients had a significantly higher ICU mortality rate of 54.07% and higher scores in SAPS II and APACHE II (Table 1). Furthermore, a logistic regression analysis revealed not only a significantly higher mortality in patients with high RDW values, but also the highest risk above the identified cut‐off value. Results of the multivariate logistic regression and COX regression analyses assessing risk factors for ICU mortality regarding RDW values at admission and the estimated cut‐off value are presented in Tables 2 and 3.

**Table 2:** Multivariate logistic regression analyses of risk factors influencing ICU mortality (for RDW cut‐off value ≥ 16.4%).

Multivariate logistic regression	*p* value	OR	95% CI
APACHE II (at admission)	< 0.0001	1.07	1.04–1.11
Age	< 0.0001	1.02	1.01–1.03
ECMO	< 0.0001	3.09	2.33–4.12
RDW cut‐off 16.4	< 0.0001	2.81	2.15–3.7

*Note:* Parameters considered in the multivariate regression models were sex (female); APACHE II at admission; ECMO (yes/no); age; RDW cut‐off, body mass index (BMI), ICU length of stay, Static compliance, SOFA score at admission, ARDS severity according to the Berlin definition. Data of 1037 patients were included. RDW% at ICU admission was used for analysis.

Abbreviations: CI, confidence interval; ECMO, extracorporeal membrane oxygenation; ICU, intensive care unit; OR, odds ratio; RDW, red cell distribution width.

**Table 3:** Cox regression analyses of risk factors influencing ICU mortality (for RDW cut‐off value ≥ 16.4%).

Cox regression	*p* value	HR	95% CI
APACHE II (at admission)	< 0.001	1.02	1.01–1.03
Age	< 0.001	1.01	1.01–1.02
ECMO	< 0.001	1.45	1.18–1.78
RDW cut‐off 16.4	< 0.001	1.96	1.61–2.4

*Note:* Parameters considered in the Cox regression models were sex (female); APACHE II at admission; ECMO (yes/no); age; RDW cut‐off, body mass index (BMI), ICU length of stay, Static compliance, SOFA score at admission, ARDS severity according to the Berlin definition. Data of 1037 patients were included. RDW% at ICU admission was used for analysis.

Abbreviations: CI, confidence interval; ECMO, extracorporeal membrane oxygenation; HR, hazard ratio; ICU, intensive care unit; RDW, red cell distribution width.

### 3.3 Secondary Endpoints

**Figure 3:**
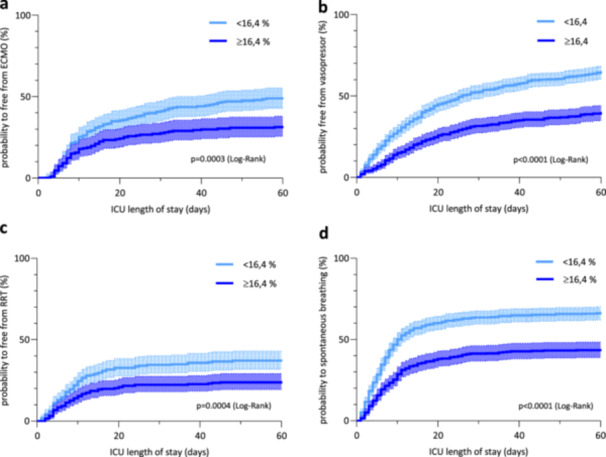
Cumulative incidence curves of probability to (a) ECMO‐free (log‐rank test, *p* = 0.0003), (b) vasopressor‐free (log‐rank test, *p* < 0.0001), (c) renal replacement therapy‐free (log‐rank test, *p* = < 0.001), (d) spontaneous breathing days composites grouped by RDW threshold ≥ 16.4% (log‐rank test, *p* < 0.0001). For each curve, the 95% CI is shown as dotted lines.

Over the investigation period, secondary endpoints, such as ECMO‐free days (log‐rank, *p* < 0.0003), vasopressor‐free days (log‐rank, *p* < 0.0001), RRT‐free days (log‐rank, *p* < 0.0004), and ventilator‐free days (log‐rank, *p* < 0.0001), were significantly different between patients above or below the respective RDW cut‐off value (Figure 3a–d).

**Figure 4:**
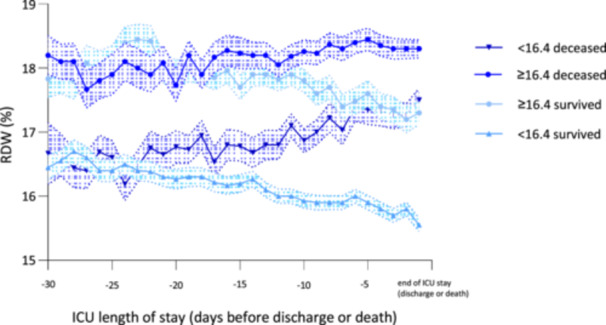
RDW value trend over the course of time grouped as indicated and by RDW cut‐off value ≥ 16.4% starting from −30 days before discharge or death.

In addition, the RDW values of patients who died showed an increase over time, regardless of whether they were in the RDW group below or above the estimated cut‐off of 16.4%. Patients who survived showed a trend toward lower RDW values (Figure 4).

## 4 Discussion

In this observational study, we evaluated the prognostic value of RDW in critically ill patients with ARDS. Our findings highlight the utility of RDW as a marker for both disease severity and mortality risk, offering implications for clinical decision‐making and patient stratification in intensive care settings. Our results demonstrate that RDW at ICU admission correlates with mortality in ARDS patients. Higher RDW values were significantly associated with increased ICU mortality, prolonged ICU stay, and worse clinical outcomes, aligning with previous studies on RDW in critically ill populations [22, 23, 24, 25]. The identified cut‐off value of ≥ 16.4% provides a clinically actionable threshold, effectively stratifying patients by risk. Notably, patients with RDW values above this threshold had nearly double the risk of ICU death compared to those with lower values. Several studies have already shown RDW as an independent predictor of higher mortality in patients with ARDS, although the study data were usually small [16, 19, 26].

These results are consistent with our findings that RDW at ICU admission predicts mortality in ARDS with moderate prognostic power. Yu et al. [16] confirmed in their propensity score‐matched cohort study that a higher RDW was associated with a higher 30‐day mortality rate, but not with longer ICU stay. In their retrospective cohort study, Sunkonkit et al. showed that an RDW value ≥ 15%, in conjunction with an elevated mean platelet volume, was an independent predictor of increased 28‐day mortality in pediatric ICU patients [27]. Furthermore, the prognostic value of other potential parameters in patients with ARDS has been investigated, but further studies are required to confirm and quantify these results [20, 28, 29]. Our results not only underscore the association between high RDW and mortality, but also show that an RDW below the threshold at ICU admission is associated with a longer ICU stay. This may be due to the higher mortality of patients who reach the estimated cut‐off value at ICU admission being associated with a shorter ICU stay. To our knowledge, RDW values were not associated with the severity of ARDS in other studies [16, 19]. We found that higher RDW values corresponded well with the severity of lung failure according to the Berlin definition, although the difference between RDW values of patients with mild and moderate was not significant. In our study data, 96.3% of 1037 patients had elevated RDW values above 14.5%, showing the critical illness of this population. This led to a cut‐off level of RDW ≥ 16.4% at ICU admission for best risk stratification, which is relatively high compared to other studies [16, 19, 30]. The elevated SAPS II and APACHE scores emphasize the severity of this critically ill patient population. Additionally, RDW values tended to increase over time in patients who eventually died, regardless of whether they were in the group below or above the estimated cutoff of 16.4%. This suggests that a progressive rise in RDW may be associated with a worsening clinical status and could serve as a dynamic prognostic marker. Surviving patients showed a trend toward lower RDW values, which could reflect the resolution of underlying inflammation, improved erythropoiesis, or overall clinical recovery (Figure 4). The findings support prior studies indicating that elevated and rising RDW levels are associated with adverse outcomes in critically ill patients, likely due to systemic inflammation's impact on red blood cell morphology, as well as the effects of oxidative stress and nutritional deficiencies [31].

Beyond mortality, in our study, RDW was associated with several secondary endpoints, including reduced vasopressor‐free days, prolonged mechanical ventilation, and delayed achievement of spontaneous breathing. These findings may emphasize the multifaceted prognostic value of RDW in ARDS, reflecting its capacity to predict organ dysfunction and the clinical course. However, RDW measurement may depend on each laboratory and may differ from place to place [12]. RDW is a routinely available, cost‐effective biomarker that can be easily integrated into clinical practice. Its prognostic utility in ARDS offers a valuable tool for risk stratification, potentially guiding decisions regarding resource allocation, escalation of care, and early referral to specialized ARDS/ECMO centers. This study established a clinically relevant RDW cutoff for risk stratification in ARDS patients at ICU admission, providing a robust foundation for future research. The study's findings encourage further multicenter, prospective investigations to validate RDW as a prognostic biomarker and to explore its integration with other clinical parameters. This integration might enhance predictive accuracy and guide the development of tailored treatment strategies in critical care.

## 5 Limitations

This study benefits from a large, well‐characterized cohort of ARDS patients. However, it is limited by its single‐center design, which may restrict generalizability. Additionally, while our findings establish an association between RDW and outcomes, causal relationships remain speculative. Variable selection was based on univariate significance and performed in Prism 10, which does not support information criteria‐based model selection. This may limit model robustness. Also, multiple statistical comparisons were performed without formal adjustment, which may increase the risk of Type I error. Therefore, the results should be interpreted with caution. Second, the study was conducted at a single high‐volume tertiary referral center, which may introduce selection bias and limit the generalizability of the findings to other ICU populations or healthcare settings. The RDW cut‐off used in this study was derived from the same data set in which it was applied. This approach may introduce bias due to overfitting and overestimation of predictive performance. As such, the results should be interpreted as exploratory and require validation in an independent cohort. Further research is warranted to explore the mechanistic pathways linking RDW to ARDS progression and to validate our findings.

## 6 Conclusions

In conclusion, our study demonstrates that RDW at ICU admission is a moderate prognostic marker for mortality and disease severity in critically ill patients with ARDS. A cut‐off value of ≥ 16.4% effectively stratifies patients by risk, with higher RDW values significantly associated with increased ICU mortality and worse clinical outcomes. Beyond mortality, RDW correlates with prolonged mechanical ventilation and reduced vasopressor‐free days, highlighting its broader clinical relevance. Due to its routine availability and cost‐effectiveness, RDW may serve as a valuable tool for risk stratification and guiding clinical decisions in the management of ARDS, perhaps in conjunction with other prognostic markers. Future multicenter studies are needed to validate these findings and further investigate the underlying mechanisms linking RDW to ARDS progression.

## Author Contributions

**Anna Kirsch:** formal analysis, visualization, writing – original draft, writing – review and editing. **Felix Niebhagen:** formal analysis, writing – original draft, writing – review and editing. **Sandra Waske:** formal analysis, visualization, writing – review and editing. **Fabian Schröer:** data curation, formal analysis, writing – review and editing. **Victoria Buenger:** data curation, formal analysis, writing – review and editing. **Oliver Hunsicker:** data curation, writing – review and editing. **Steffen Weber‐Carstens:** data curation, formal analysis, writing – review and editing. **Jan Adriaan Graw:** data curation, formal analysis, writing – review and editing. **Mario Menk:** conceptualization, data curation, formal analysis, methodology, resources, supervision, validation, visualization, writing – original draft, writing – review and editing. All authors have read and approved the final version of the manuscript.

## Consent

The authors have nothing to report.

## Conflicts of Interest

The authors declare no conflicts of interest.

## Transparency Statement

The lead author, Mario Menk, affirms that this manuscript is an honest, accurate, and transparent account of the study being reported; that no important aspects of the study have been omitted; and that any discrepancies from the study as planned (and, if relevant, registered) have been explained.

## Data Availability

The data associated with the paper are not publicly available but are available from the corresponding author on reasonable request. M.M. organized the study as an overall supervisor and had full access to all of the data in this study and takes complete responsibility for the integrity of the data and the accuracy of the data analysis.
